# Recent advances in understanding female gametophyte development

**DOI:** 10.12688/f1000research.14508.1

**Published:** 2018-06-20

**Authors:** Debra J Skinner, Venkatesan Sundaresan

**Affiliations:** 1 Department of Plant Biology, University of California-Davis, Davis, USA; 2 Department of Plant Sciences, University of California-Davis, Davis, USA

**Keywords:** female gametophyte, embryo sac

## Abstract

The haploid female gametophyte (embryo sac) is an essential reproductive unit of flowering plants, usually comprising four specialized cell types, including the female gametes (egg cell and central cell). The differentiation of these cells relies on spatial signals which pattern the gametophyte along a proximal-distal axis, but the molecular and genetic mechanisms by which cell identities are determined in the embryo sac have long been a mystery. Recent identification of key genes for cell fate specification and their relationship to hormonal signaling pathways that act on positional cues has provided new insights into these processes. A model for differentiation can be devised with egg cell fate as a default state of the female gametophyte and with other cell types specified by the action of spatially regulated factors. Cell-to-cell communication within the gametophyte is also important for maintaining cell identity as well as facilitating fertilization of the female gametes by the male gametes (sperm cells).

## Background

**Figure 1.**
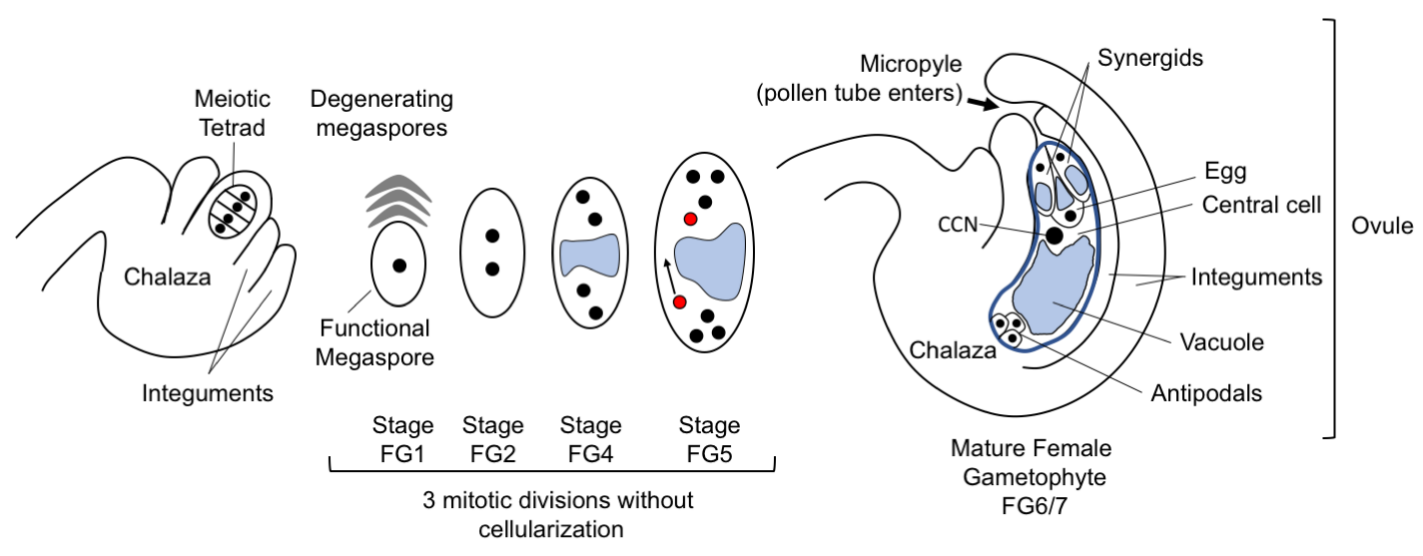
Arabidopsis female gametophyte development. The progression of female gametophyte development is shown from left to right. After meiosis, a single haploid cell, usually the basal (chalazal) cell, will enlarge and form the functional megaspore while the remaining products of meiosis degenerate. This haploid megaspore will have three mitotic divisions accompanied by nuclear movement to create a defined pattern at each division. From stage FG4, the large vacuole (blue) separates the nuclei along the chalazal-micropylar axis. At FG5, the polar nuclei (red) migrate to meet each other and eventually fuse. At FG6/FG7, the mature female gametophyte has seven cells: two synergids, egg cell, central cell with large diploid nucleus (central cell nucleus, or CCN), and three antipodal cells (which are present through FG7 though much diminished ^8^). Stages are numbered in accordance with Christensen *et al*. ^4^.

The flowering plants that dominate our landscapes and agriculture alternate between a diploid sporophytic stage, which constitutes the main body of the plant, and a reduced, haploid gametophytic stage contained within the male and female floral organs. The pollen grain is the mature male gametophyte and carries two sperm cells (the male gametes). The female gametophyte (FG), called the embryo sac, produces the female gametes and usually is obscured within the maternal, sporophytic ovule ( Figure 1). Fusion of male and female gametes occurs during double fertilization, after the sperm cells are delivered to the embryo sac by the pollen tube. After fertilization, ovules become seeds and sexual reproduction is achieved. Despite its reduced size relative to the diploid sporophyte, the development of the FG is tightly regulated as it is essential for successful seed formation. FG development in flowering plants begins after meiosis, when one of four haploid daughter cells develops into the functional megaspore (FM). In most flowering plants (including Arabidopsis), the FM undergoes three rounds of syncytial mitotic divisions, followed by cellularization to produce seven cells belonging to four cell types, each with a defined position, morphology, and specialized function ( Figure 1, 1). Two FG cell types are gametic: the egg cell (1n) and the central cell (2n, homodiploid). These undergo double fertilization by two sperm cells of the entering pollen tube to produce the embryo (2n) and endosperm (3n), respectively. There are two accessory cell types: the two synergids, whose main function is pollen tube attraction and reception by the gametophyte, and the three antipodals, whose function in many plants is currently unknown. In grasses such as maize, the antipodals proliferate further and are proposed to have a role in directing nutrition from sporophyte to developing endosperm ^2, 3^. These four cell types are specified from the eight haploid nuclei that have descended from the FM. After the first mitotic division of the FM (stage FG2, 4), the two daughter nuclei are physically sequestered at either end of the embryo sac by the enlarging vacuole, creating a morphological axis (FG3). After two further divisions (FG5), one of the four nuclei at each end migrates around the central vacuole toward the center; these polar nuclei become closely associated and will finally fuse, forming the central cell nucleus (FG6). At the same time, the remaining nuclei begin to differentiate by cellularization according to their position along the distal (micropylar)-proximal (chalazal) axis. At maturity, the pollen tube enters the ovule through the micropyle, formed by the tips of enclosing maternal integuments.

At the micropylar end of the gametophyte, the synergid cells and egg cell are in close proximity but have different morphologies, including nuclear position (the smaller synergid nuclei are oriented closer to the micropyle and egg nucleus toward the central cell) and vacuole position. Their molecular differences have been analyzed in RNA profiling experiments and these differences reflect their different roles in the FG ^5, 6^. How and when do the nuclei and the resulting cells of the gametophyte acquire fate information? What external signals are required, and how do cells communicate with each other to define or confirm these fates? These questions have been tackled by assessing mutants or ectopic expression lines which alter cell identities, observed by morphological changes, and monitored with the use of fluorescent or colorimetric reporter genes expressed in specific cell types (as reviewed in 7). Complete cell fate change is shown by change in function, such as a synergid acquiring the ability to form a zygote after fertilization (synergid to egg cell) or an egg cell to form an endosperm (egg cell to central cell). An overarching theme is that positional information is important for cell identity, as first described using the maize *indeterminate gametophyte 1* mutant ^9, 10^, and these positional cues interact with external and internal signals to specify the different cell fates.

## Specification of micropylar (distal) cell fates

**Figure 2.**
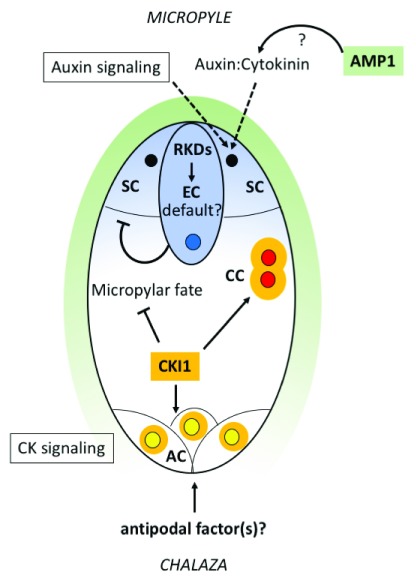
Model for acquisition of cell identity in the female gametophyte. *RKD* genes (expression pattern shown in blue) may act early to set up a default egg cell state in the gametophyte and continue to promote egg cell identity later. At the micropylar pole, auxin signaling, together with sporophytically active *AMP1* (green) which could potentially affect the auxin:cytokinin balance, acts to specify synergid cell identity. The egg cell (with blue nucleus) maintains synergid identity by suppressing egg cell fate in the adjacent synergid cells (black nuclei). *CKI1* (orange) represses micropylar fates in the chalazal domain and is needed to specify central cell identity (polar nuclei in red) in a pathway involving AHP proteins. *CKI1*, together with additional factors that may be provided from the chalaza, specifies antipodal cell fates (yellow nuclei). Note: The nuclei in this sketch are not drawn to scale. AC, antipodal cell; AHP, Arabidopsis phosphotransfer protein; AMP1, *altered meristem program 1*; CC, central cell; CK, cytokinin; CKI1, *CYTOKININ INSENSITIVE 1*; EC, egg cell; RKD, *RWP-RK DOMAIN CONTAINING*; SC, synergid cell.

Live imaging of developing *Torenia fournieri* gametophytes shows that at late FG4 (four-nucleate stage), when there are two nuclei at the micropylar end, these nuclei have a polar arrangement along the micropylar-chalazal axis ^11^. The nucleus closest to the micropyle was observed to give rise to the synergids, while the nucleus closest to the central vacuole gave rise to egg and polar nucleus. This arrangement of FG4 nuclei has also been observed in fixed Arabidopsis ovules ^12^. Differing cell fates of sister nuclei may result from asymmetric distribution of a molecular signal within the embryo sac or from an asymmetric external sporophytic signal. There are multiple lines of evidence for the importance of mobile signals in FG patterning at the micropylar pole where the egg cells and synergid cells are located ( Figure 2). The plant hormone auxin is a small mobile molecule whose synthesis and polar movement through plants direct growth and patterning decisions. Loss of synergid identity and occasional acquisition of egg identity were observed when auxin signaling genes— *TRANSPORT INHIBITOR RESPONSE* ( *TIR*) family and *AUXIN RESPONSE FACTOR* ( *ARF*) family—were downregulated or inactivated in the early developing embryo sac ^13– 15^. Conversely, ectopic expression of the auxin biosynthesis *YUCCA* genes appears to shift micropylar cell fates toward the chalazal end of the gametophyte, conferring synergid and egg cell marker expression onto the central cell and antipodal cells ^14^. Despite differing conclusions about the presence of auxin inside the embryo sac, studies are in agreement that auxin accumulation occurs in the adjacent sporophytic cells of the nucellus at the micropylar end during gametogenesis, consistent with localization of PIN and AUX1 auxin transporters in the sporophyte and gametophyte, respectively ^13– 17^. That auxin either directly or indirectly acts as a signaling molecule for micropylar specification, in particular for synergid cell fate, is also supported by the phenotypes of *yucca* mutants, in which synergids exhibit egg cell attributes ^14^.

In the *altered meristem program 1* ( *amp1*) mutant, synergid cells are converted to functional egg cells at high frequency ^18^, among other pleiotropic effects. Interestingly, sporophytic expression of *AMP1* is sufficient to rescue this phenotype. *AMP1* is detected only in the integuments early in gametophyte development and in synergids after cellularization. Therefore, AMP1 appears to mediate a mobile signal that promotes synergid identity, and in the absence of that early signal, egg cell fate is adopted. AMP1 protein is a membrane-anchored carboxypeptidase protein localized to the rough endoplasmic reticulum (ER) ^19^. The protein has been associated with translation repression by microRNAs (miRNAs) ^20, 21^ and is also important to repress biosynthesis of cytokinins ^22^. Crosstalk between cytokinin and auxin affects developmental modules in many parts of the plant; in many cases, the balance between these hormones is essential for correct patterning of cell types (reviewed in 23). It is possible that a specific ratio of auxin and cytokinin activity is needed for correct micropylar patterning and that AMP1 is necessary to maintain this balance ( Figure 2). Detailed molecular characterization of AMP1 function in the ovule may shed light on the mechanism controlling synergid specification in gametophyte development.

After cellularization, the synergid and egg cells presumably have acquired cell identity information. Despite this, cell ablation experiments in Arabidopsis and Torenia consistently show that loss of the egg cell causes morphological and marker line changes in at least one synergid, which takes on features of an egg cell and may even be fertilized ^11, 24^. Similarly, mutants in which important cellular functions of the egg cell are disrupted also cause at least partial alteration of synergid identity ^25, 26^. This suggests that the egg cell prevents its synergid neighbors from acquiring egg cell fate later in development through cell-to-cell communication ( Figure 2). In contrast, in the ablation experiments, the central cell is not disrupted, can be fertilized, and does not take on aspects of egg cell morphology ^24^, indicating that polar nuclei and the central cell are not subject to the same interaction.

## Specification of chalazal (proximal) cell fates

A key differentiation factor for the nuclei at the chalazal end of the FG is *CYTOKININ INSENSITIVE 1* ( *CKI1*), an ER-localized histidine kinase that can activate cytokinin responses constitutively ^27– 29^. *cki1* mutants show loss of central cell and antipodal identities and expansion of egg cell and, in some cases, synergid attributes, suggesting that CKI1 suppresses micropylar cell fates ^29– 31^. *CKI1* expression, initially present at both poles through FG3, is quickly restricted to the two nuclei of the chalazal end at FG4. This polarity of expression is maintained through the next nuclear division (FG5 stage). After the chalazal polar nucleus and its associated ER migrate toward the micropylar polar nucleus at stage FG5, *CKI1* expression continues in the resulting diploid cell as well as in the antipodal cells at the chalazal end. Ectopic expression of *CKI1* is sufficient to induce central cell fate in the egg cell and synergids and produce seeds with multiple ectopic endosperms but lacking embryos. Therefore, *CKI1* appears to specify central cell identity while restricting micropylar cell fates ( Figure 2). Similar to the cytokinin receptors, *CKI1* acts through activation of a two-component signaling cascade, involving phosphorylation of Arabidopsis phosphotransfer proteins (AHPs), which then activate downstream transcription factors. Specifically, in the FG, AHP2, 3, and 5 are required for CKI1 function ^32^. Transcription factors that are potential targets of this pathway include the MADS box–containing genes *AGL80* and *DIANA*/ *AGL61*, which are known regulators of central cell–specific pathways ^33– 35^. Transcription factor *MYB119* is also a likely target of CKI1 regulation and acts redundantly with *MYB64* to promote cellularization ^36^. *myb64 myb119* mutants fail to cellularize and they show continued nuclear divisions and an expansion of central and antipodal cell fates. It is likely that the absence of cell membranes allows expansion of central cell and antipodal cell identity factors and suggests that *CKI1* not only promotes chalazal identity but also a mechanism to limit that identity to the appropriate space.

*CKI1* is expressed in antipodal cells and is required for antipodal cell specification, as antipodal cells acquire egg cell attributes in *cki1* mutants. At the same time, *CKI1* does not alter antipodal cell fate when overexpressed; that is, ** antipodal cells are not re-specified as central cells ^29^. This suggests that *CKI1* action must be redirected in these cells by an additional antipodal specification factor acting at the chalazal end ( Figure 2). Such a factor could be supplied by chalazal sporophytic cells, as the antipodal cells are in close contact with this tissue. Movement of a fluorescent protein (ZsYellow) from antipodals to neighboring maternal cells has been demonstrated ^24^, suggesting a symplastic connection between these cells that may allow movement of an identity signal. In Arabidopsis, the antipodal cells become inconspicuous and eventually degenerate after fertilization ^8^, but in maize and other grasses, the antipodal cells proliferate instead of diminishing, perhaps to facilitate nutrient transfer from the sporophyte to developing endosperm and embryo ^2, 3^. Proliferation of the antipodals in maize has been proposed to involve auxin signaling ^37^. Maintenance of antipodal identity in the proliferating antipodal cells requires a secreted, grass-specific factor, ZmEAL1, that is synthesized in the egg cell; without this factor, the antipodals acquire central cell characteristics at low frequency ^38^. Orthologs of ZmEAL1 can be found in other grasses but not in eudicots. In summary, in both Arabidopsis and maize, specification of antipodals requires additional factors but these factors are likely to be different, as suggested by the very different fate of the antipodal cells in grasses.

## Egg cell as the default state?

Egg cell fate predominates in the absence of *CKI1*, as *cki1* mutants fail to specify antipodals and central cells, and nuclei at the chalazal positions express egg cell markers instead ^29^. Similarly, at the micropylar positions, there are a number of different mutants—such as *amp1*, *eostre*, *lachesis*, and *yucca1 yucca2*—in which synergids are not correctly specified and acquire egg cell fates instead ^14, 18, 25, 39^. These phenotypes raise the possibility that egg cell identity is a “default” state in the FG and that additional spatially regulated factors are required to specify the other cell types. Recent research has suggested candidate factors that may set up such a default state. Members of the plant-specific *RWP-RK DOMAIN CONTAINING* ( *RKD*) gene family have been suggested as egg cell determinants because of their high expression in egg cells and ability to activate egg-like transcription profiles when ectopically expressed ^24, 40, 41^. In addition, loss of the putatively orthologous single-copy gene in *Marchantia polymorpha*, *MpRKD*, leads to failure to develop mature egg cells ^42, 43^. Thus, *RKD* genes seem to have a conserved role in egg cell differentiation. However, recent examination of the *RKD* gene family has suggested a complex role in Arabidopsis. Thus far, only *RKD2* was shown to be specific to mature egg cells, but other family members are expressed in egg and other cells of the gametophyte ^5, 6, 40, 44^. *RKD1* and *2* are capable of activating aspects of the egg cell transcriptome ectopically in protoplasts, callus, and sporophytic cells of the ovule ^24, 40^. These overexpression phenotypes suggested that *RKD1* and *RKD2* are activators of egg cell identity, but neither the single mutants nor a double mutant led to obvious changes in cell type in the gametophyte ^40, 44^. Investigating redundancy with other gene family members showed that *RKD2* acts from the earliest stages of FG development, as double mutants with *RKD4* and *RKD5* cause significant FM arrest as well as loss of egg cell identity markers ^44^. Taken together, *RKD* genes of Arabidopsis seem to act redundantly at multiple stages of reproductive development, including proper progression from FM to FG, and in activation of egg cell differentiation. Considering their early action in the FG and ectopic expression results, we suggest that *RKD* genes may help to create a default egg cell–like state in the developing FG and may continue to be important for egg cell differentiation later. In this scheme, differentiation into cell types other than the egg cell would then require additional cell factors (for example, *CKI1* and *AMP1*) whose actions are governed by nuclear positions at cellularization.

## Double fertilization requires multiple cell types to be specified in the female gametophyte

The correct specification of cell fates is important not only for formation of the female gametes but also for double fertilization, a process that requires the participation of three of the four cell types in the FG. This process has been reviewed extensively in recent articles ^45, 46^, so we present only a brief outline here. A complex set of pollen attractants, and structural changes to cell membranes are produced by synergid cells, regulated in part by specific expression of *MYB98* in mature synergids ^47– 49^. Recently, central cell transcription factors *CENTRAL CELL GUIDANCE* ( *CCG*) and *CCG BINDING PROTEIN 1* were shown to interact with each other and other binding partners to influence expression of *MYB98* as well as expression of small mobile peptides that themselves may be pollen tube attractants ^50, 51^. After entry of the pollen tube, fusion of sperm with egg and central cell requires egg cell–specific secreted peptides of the *EGG CELL 1* ( *EC1*) family ^52, 53^. Recent evidence involves chromatin remodeling factors and the transcription factor *SUPPRESSOR OF FRIGIDA 4* in regulation of *EC1* genes ^54^. Pollen tube reception destroys one synergid, leaving the other intact and capable of attracting another pollen tube, but only if the gamete fusions from the initial pollen tube fail ^55– 57^. Sperm cell and egg cell fusion signals successful fertilization via ethylene signaling ^58^, and the newly fertilized central cell (now endosperm) fuses with the remaining synergid, thereby diluting its ability to attract pollen tubes ^59^. Central cell–specific Polycomb repressive complex genes are required for this fusion, indicating that proper specification of the central cell is one essential aspect of this process. In summary, successful double fertilization requires multiple processes and relies on specific gametophytic cell types. Pollen tube guidance is provided primarily by the synergids but also the central cell while pollen tube entry occurs through the synergid cells. Sperm cell fusion with the two female gametes requires factors provided by the egg cell; finally, suppression of the pollen tube attraction signal occurs by fusion of the central cell with the persistent synergid cell. Thus, successful double fertilization to produce a seed is an orchestrated process involving multiple interactions of the synergid cells, the egg cell, and the central cell. As discussed in this review, the overall themes of how these different cell types are specified are emerging gradually from the multiple approaches employed by several different laboratories. However, the molecular details are still rudimentary as compared with those of other developmental processes such as floral or meristem development, and there is a vital need for further elucidation of the relevant developmental pathways in the FG, given its critical importance to flowering plant reproduction.

## Abbreviations

AHP, Arabidopsis phosphotransfer protein; amp1, *altered meristem program 1*; CCG, *CENTRAL CELL GUIDANCE*; CKI1, *CYTOKININ INSENSITIVE 1*; EC1, *EGG CELL 1*; ER, endoplasmic reticulum; FG, female gametophyte; FM, functional megaspore; RKD, *RWP-RK DOMAIN CONTAINING*.
